# Variation in the management of herpes zoster among US nursing home residents with and without dementia

**DOI:** 10.1093/geroni/igaf122.2862

**Published:** 2025-12-31

**Authors:** Peyton Free, Preeti Chachlani, Kaleen Hayes, Kevin McConeghy, H Edward Davidson, Lisa Han, Stefan Gravenstein, Daniel Harris

**Affiliations:** University of Delaware, Newark, Delaware, United States; Brown University School of Public Health, Providence, Rhode Island, United States; Brown University, Providence, Rhode Island, United States; Brown University School of Public Health, Providence, Rhode Island, United States; Insight Therapeutics, Norfolk, Virginia, United States; Insight Therapeutics, Norfolk, Virginia, United States; Alpert Medical School of Brown University, Providence, Rhode Island, United States; University of Delaware, Newark, Delaware, United States

## Abstract

Herpes zoster (HZ) is a painful condition that is managed with a combination of antivirals, steroids, and pain medications. Nursing home residents with Alzheimer’s disease and related dementias (ADRD) may have difficulty expressing HZ symptoms, thus affecting medication management. This study compared prescription and non-prescription medication use in nursing home residents with HZ by ADRD diagnosis. We conducted a retrospective cohort study using nursing home electronic health record data from 2017-2022. Residents with HZ and ADRD were identified using ICD-10 codes as of the HZ diagnosis date. Administration for pain medications, antivirals, and steroids were measured before and after the HZ diagnosis date in periods of time (e.g., 1-7, 8-14, and 15-30 days before/after diagnosis). To assess relative changes in medication prevalence over time, administrations 15-30 days prior to HZ represented the baseline period. We identified 4,719 residents with HZ (31.5% had ADRD). Among residents without ADRD, oxycodone increased from 1.6% to 3.4% (vs. ADRD=0.5% to 0.8%) and gabapentin from 13.4% to 33.9% (vs. ADRD=12.6% to 25.6%) between baseline and 0-7 days after HZ diagnosis. In the 0-7 days after HZ diagnosis, prednisone use was 1.74 times greater in residents without ADRD (10.3%) vs. with ADRD (5.9%), and acyclovir use was similar between groups (without ADRD=28.5%; with ADRD=27.1%). Differences in medications, especially prednisone, between residents with and without ADRD may reflect undertreatment of HZ symptoms in residents with ADRD.

